# Identification of Genomic Insertion and Flanking Sequence of *G2-EPSPS* and *GAT* Transgenes in Soybean Using Whole Genome Sequencing Method

**DOI:** 10.3389/fpls.2016.01009

**Published:** 2016-07-12

**Authors:** Bingfu Guo, Yong Guo, Huilong Hong, Li-Juan Qiu

**Affiliations:** ^1^The National Key Facility for Crop Gene Resources and Genetic Improvement (NFCRI) and MOA Key Lab of Soybean Biology (Beijing), Institute of Crop Science, Chinese Academy of Agricultural SciencesBeijing, China; ^2^College of Agriculture, Northeast Agricultural UniversityHarbin, China

**Keywords:** insertion site, flanking sequence, whole genome sequencing, transgenic soybean, next generation sequencing

## Abstract

Molecular characterization of sequence flanking exogenous fragment insertion is essential for safety assessment and labeling of genetically modified organism (GMO). In this study, the T-DNA insertion sites and flanking sequences were identified in two newly developed transgenic glyphosate-tolerant soybeans GE-J16 and ZH10-6 based on whole genome sequencing (WGS) method. More than 22.4 Gb sequence data (∼21 × coverage) for each line was generated on Illumina HiSeq 2500 platform. The junction reads mapped to boundaries of T-DNA and flanking sequences in these two events were identified by comparing all sequencing reads with soybean reference genome and sequence of transgenic vector. The putative insertion loci and flanking sequences were further confirmed by PCR amplification, Sanger sequencing, and co-segregation analysis. All these analyses supported that exogenous T-DNA fragments were integrated in positions of Chr19: 50543767–50543792 and Chr17: 7980527–7980541 in these two transgenic lines. Identification of genomic insertion sites of *G2-EPSPS* and *GAT* transgenes will facilitate the utilization of their glyphosate-tolerant traits in soybean breeding program. These results also demonstrated that WGS was a cost-effective and rapid method for identifying sites of T-DNA insertions and flanking sequences in soybean.

## Introduction

Genetically modified crops (GM crops) were first commercialized in 1996 and since then they have been grown and consumed for two decades. During this period, a large number of transgenic plants have been developed and released (Liang et al., 2014). Up to now, the cumulative hectarage of biotech crops has exceeded two billion hectares globally (James, 2015), and more and more foods and feeds derived from GM plants have been entering into supply chains. In addition, a growing number of genes or regulatory elements have still been transferred into crop genomes to improve agronomic traits (Daniela et al., 2013). Once transgenic lines showing excellent agronomic performance are generated, the extensive testing and comprehensive analyses of these lines are necessary for biosafety assessment before being approved and entering into market (Codex Alimentarius Commission, 2003; European Food Safety Authority [EFSA], 2010; Kok et al., 2015). Among these, low copy number integration is the most favorable molecular profile for selecting the best events from putative lines (Kovalic et al., 2012). Furthermore, development of even-specific detection methods is not only useful for breeding program, but also of particular importance for bio-risk management to ensure food, feed, and environmental safety (Arne et al., 2012; Daniela et al., 2013; Fraiture et al., 2015a).

Traditionally, T-DNA flanking sequence of transgenic plant is identified by using PCR-based methods. These methods include thermal asymmetric interlaced PCR (TAIL-PCR; Liu et al., 1995), adapter-ligated PCR (O’Malley and Ecker, 2010), inverse PCR (Ochman et al., 1988), or restriction site extension PCR (Ji and Braam, 2010), which all rely on the sequence information of transgenic elements (Spalinskas et al., 2013). Among these, TAIL-PCR and genome walking are commonly used approaches for isolating and cloning sequences flanking T-DNA (Daniela et al., 2013). Several junction sequences in transgenic soybean, maize, and cotton were successfully characterized using these methods (Windels et al., 2001; Yang et al., 2005; Akritidis et al., 2008; Wang et al., 2010; Fraiture et al., 2015b). However, these approaches are always laborious and expensive, and especially difficult to achieve high throughput. Even more, if the genome of plant species is complex or the transgenic event contains intricate modifications or rearrangements of exogenous fragment, these traditional methods are not powerful enough to identify all insertion loci and their flanking sequences (Daniela et al., 2013).

With the emergence and fast development of NGS technologies, sequence from whole genome can be generated in a short time with a low cost. NGS approaches have been proven to be powerful tools for discovering gene fusions, sequence rearrangements, DNA insertions, and structural variations in different animal and plant species (Campbell et al., 2008; Fullwood et al., 2009; Hormozdiari et al., 2011; Kovalic et al., 2012; DuBose et al., 2013). For the past few years, NGS has also provided an alternative tool in molecular characteristics of GM plants. Several NGS based methods have been developed to identify insertions of exogenous fragments in *Arabidopsis thaliana* (Lepage et al., 2013; Inagaki et al., 2015), rice (Daniela et al., 2013; Park et al., 2015), and maize (Rosalind et al., 2010). Compared with PCR-based methods, combination of targeted bioinformatics analysis and limited *de novo* assembly using WGS data has become a much simpler and more effective approach for transgenic analysis.

Soybean is a paleopolyploid species with nearly 75% of genes presented in multiple copies due to the lack of immediate diploidization during the relatively recent whole genome duplication (Kim et al., 2009). Two rounds of genome duplication occurring at approximate 59 and 13 million years ago result in a highly duplicated genome and numerous chromosome rearrangements (Schmutz et al., 2010). Therefore, traditional PCR-based methods are always failed to identify insertion sites in GM soybean. The complete sequence of soybean cultivar Williams 82 provides a reference for whole genome re-sequencing and genomics research of different soybean genotypes (Schmutz et al., 2010). Like other model plants, NGS method has been proved to be successful in examining typical GM soybean lines MON17903 and MON87704 whose insertion sites and flanking sequences had been identified previously (Kovalic et al., 2012). However, whether it can still be efficient for molecular characterization of uncharacterized transgenic lines remains unclear.

Among all commercialized GM crops, herbicide tolerant transgenic soybean has been the most widely grown one all over the world. Recently, we developed two transgenic lines GE-J16 and ZH10-6 by co-expression of glyphosate tolerant gene *G2-EPSPS* and glyphosate-degrading gene *GAT*, which conferred high tolerance to the herbicide glyphosate in soybean (Guo et al., 2015a,b). In this study, the integration sites and junction sequences of *G2-EPSPS* and *GAT* transgenes were characterized from these two events using WGS method. The reads mapped to junctions of T-DNA and host genomes of them were selected by bioinformatics analysis and putative integration sites were identified. The exact insertion sites and flanking sequences were further determined after validation by PCR amplification and Sanger sequencing. Molecular characterization of these two herbicide tolerant transgenic soybeans at nucleic acid level will provide precise information for regulatory submissions and facilitate utilization of these soybean lines in future breeding program.

## Materials and Methods

### Plant Materials

The transgenic soybeans GE-J16 and ZH10-6 were produced by *Agrobacterium*-mediated transformation of soybean cultivars Jack and ZH10 (Guo et al., 2015a,b). The plasmid vector pKT-rGE used for transformation contains glyphosate tolerant gene *G2-EPSPS* and glyphosate-degrading gene *GAT*. The transformants co-expressing *G2-EPSPS* and *GAT* genes conferred high tolerance to glyphosate. Southern blot analysis indicated that only one copy of exogenous T-DNA was integrated into each host genome (Guo et al., 2015a,b).

### Genetic Analysis of GM Soybean Events

T_2_ progeny derived from heterozygous lines of GE-J16 and F_2_ populations developed by crossing between homozygous ZH10-6 and non-transgenic soybean cultivars (HH38, HH43, KS1, KF16, KF20 and KF22) were used for genetic analysis. Soybean plants were sprayed with commercial formulation of glyphosate (Roundup, Monsanto Co.) at the labeled rate (1800 g a.e./ha) when first trifoliolate leaves were fully expanded. The number of living and dead plants was investigated two weeks after treatment and segregation ratios were analyzed by χ^2^ testing. The data was analyzed using SPSS 18.0 and Excel.

### Genomic DNA Isolation and Whole Genome Sequencing

Genomic DNA was isolated from fresh leaves of soybean plants using the modified CTAB method (Porebski et al., 1997) and quantified by Quawell Q5000 spectrophotometer (Quawell Technology, Inc., USA). About 5 μg of genomic DNA from GE-J16 and ZH10-6 was sheared to fragments with a length of 400 bp in average to construct libraries using the Nextera DNA Sample Preparation Kit (Illumina, USA). The libraries were then subjected to sequencing on Illumina Hiseq2500 platform and 125-bp paired-end reads were generated.

### Transgenic Insertion Analysis

**FIGURE 1:**
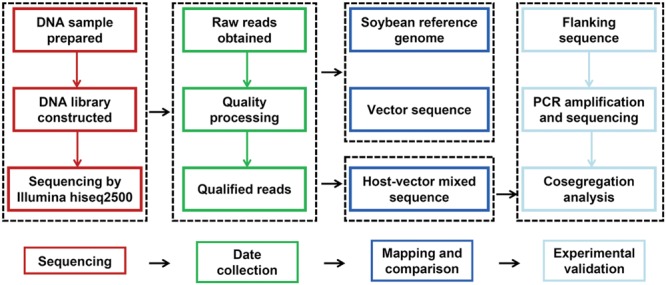
**Schematic diagram of the work-flow for insertion site identification and validation**.

Data obtained from the sequencer was processed for quality control and raw reads were filtered by removal of adapter and low quality reads (*Q* < 20). Clean reads were individually aligned and mapped to *Glycine max* Wm82.a2.v1 reference genome from Phytozome and sequence of pKT-rGE vector using BWA with default parameters (Langmead and Salzberg, 2012). The pipeline for data analysis and validation was briefly described in **Figure 1**. After mapping of all reads against the reference genome and sequence of vector, they were classified into three groups: reads only mapped to the reference genome, reads only mapped to vector sequence and reads mapped to both sequences (junction reads). Physical positions of junction reads were indicated the integration sites and were used for further analysis.

### Confirmation of Insert Loci and Flanking Sequences by PCR and Sanger Sequencing

The upstream and downstream sequences flanking putative insertion sites identified by junction reads were extracted from soybean genome database at Phytozome^1^. For each transgenic soybean line, a total of four primers were designed based on putative flanking sequences and T-DNA sequence. Two primers were annealed within upstream and downstream flanking sequences and the other two annealed to exogenous *G2-EPSPS* and *GAT* in T-DNA region. One primer binding putative flanking sequences and the other binding T-DNA region were used in combination to amplify the putative junction sequences. PCR products were checked on 1% agarose gel by electrophoresis and specific bands were sequenced on both strands. Sequence alignment was performed to identify exact insertion positions of exogenous fragments. The primers used for amplification were listed in **Table 1**

**Table 1:** Event specific primers used in this study.

Primer name	Sequence (5′ to 3′)	Product size (bp)
JackP-1	CAGCTAAAGATATAGTGTCAAGAACCT	1529
GAT-1	GCGATTTACTTCGTGGTGCAT	
G2EP-1	ACCACCATCAATCTCGAAACG	2203
JackP-2	CAATTCAAGACAGAAAATACGATGA	
ZH10P-1	TAATAGTAGAATGGGACTGGTGGAT	810
GAT-2	GCGGACTTGCTTTGGTGTAAT	
G2EP-2	CCCGAATCATCAGGCAAACA	1626
ZH10P-2	AACACATCATAGTATTCTAAAACGCTT	

### Validation of Integration Sites in Segregation Populations

The event-specific primer pairs were applied to amplify the progeny of heterozygous lines and segregation populations. PCR amplification was carried out in 20-μl reaction mixture using PTC-200 Thermocycler (MJ Research/Bio-Rad, USA). The PCR procedures were as follows: 1 cycle of 94°C for 4 min; 36 cycles of 94°C for 30 s, 60°C for 30 s, and 72°C for 90 s; with a final extension of 72°C for 10 min. PCR products were analyzed on 1% agarose gel by electrophoresis.

## Results

### Genetic Analysis of Transgenic Soybean Lines

In order to identify segregation ratios of exogenous genes in GM soybeans, progeny of three heterozygous lines of GE-J16 and six F_2_ populations derived from ZH10-6 were used for phenotype identification and genetic analysis. After spraying with herbicide Roundup, the number of living and dead plants in each population was counted. The results showed that observed ratios of glyphosate tolerante and sensitive plants in these populations were all well fitted to 3:1 ratio with χ^2^ values range from 0 to 1.922 (**Table 2**). PCR amplifications of exogenous genes also suggested the existance of them co-segragated with the tolerance of glyphosate (data not shown). These results further confirmed that one insertion site of exogenous gene was intergrated into the genome of each transgenic event.

**Table 2:** Genetic analysis of heterozygosis lines of GE-J16 and F_2_ populations derived from ZH10-6.

Types of populations	Names of populations	Total No. of plants treated	No. of tolerant plants	No. of sensitive plants	Observation ratio	χ^2^ (3:1)	*P*-value
Heterozygosis lines of GE-J16	GE-J16-1	126	98	28	3.50:1	0.519	0.471
	GE-J16-2	136	95	41	2.32:1	1.922	0.166
	GE-J16-4	117	92	25	3.68:1	0.823	0.364
F_2_ populations derived from ZH10-6	HH43 × ZH10-6	136	103	33	3.12:1	0.039	0.843
	KS1 × ZH10-6	118	84	34	2.47:1	0.915	0.339
	HH38 × ZH10-6	261	194	67	2.90:1	0.063	0.802
	KF22 × ZH10-6	103	81	22	3.68:1	0.728	0.393
	KF16 × ZH10-6	64	48	16	3.00:1	0.000	1.000
	KF20 × ZH10-6	93	68	25	2.72:1	0.176	0.675

*P-value > 0.05 or χ^2^ (3:1) < 3.841 indicated that segregation ratio was consistent with the 3:1 ratio.*

### Whole Genome Sequencing of GM Soybean Events

Whole Genome Sequencing was used for identifying molecular characterizations of GM soybeans and major steps were described in **Figure 1** Sequencing libraries were constructed and sequencing reads in length of 125-bp were generated by paired-end sequencing. After the processing of quality control, a total of 179.3 million clean reads for GE-J16 and 210.0 million clean reads for ZH10-6 were obtained. Among them, 90.11% and 87.96% of sequencing data has Phred-like quality scores ≥30 (**Table 3**), indicating the high quality of the data. About 95.38% and 93.25% reads could map to soybean reference genome in these two soybean lines, accounting for ∼20 × and ∼22 × coverage of soybean genome, respectively. Among them, about 92.6% of genome had at least one-fold coverage and nearly half of genome had at least ten-fold coverage (**Table 3**).

**Table 3:** The summary of sequence data from WGS.

Transgenic events	GE-J16	ZH10-6
Clean reads	179,326,462	210,087,270
Clean bases (Gb)	22.41	26.26
Q20(%)	96.80	92.86
Q30(%)	90.11	87.96
Mapped ratio(%)	95.38	93.25
Average depth	20	22
Coverage_ratio_1x(%)	92.58	92.59
Coverage_ratio_5x(%)	74.03	76.27
Coverage_ratio_10x(%)	48.45	51.80

### Identification of Putative Integration Sites Using Whole Genome Sequencing Data

**FIGURE 2:**
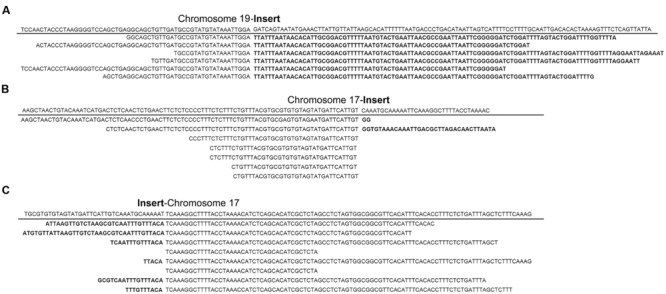
**Junction reads covering junctions of integrated T-DNA and host genomes in GE-J16 and ZH10-6**. Junctions with transition on insert and chromosome 19 in GE-J16 **(A)** and transition on insert and chromosome 17 in ZH10-6 **(B,C)**. The sequence of reference genome along the insertion site was underlined and the transition between soybean genome and T-DNA insertion was indicated by a gap. The part of each read belonging to the exogenous fragment was indicated in bold.

In order to identify putative insertion sites of exogenous fragments, all clean reads were mapped to the sequence of pKT-rGE vector and soybean reference genome. The putative integration sites of transgenic events were characterized based on junction reads in which one end was mapped to the sequence of vector and the other end to the host genome. After detailed data analysis, six junction reads on chromosome 19 and 15 reads on chromosome 17 were identified from the sequence data of GE-J16 and ZH10-6 separately (**Figure 2**). According to physical positions of junction reads, the T-DNA is integrated at position around Chr19: 50,543,500-50,543,900 in GE-J16 and the insertion loci of ZH10-6 was located at position Chr17: 7,980,300-7,980,600. These results further confirmed a single insertion site of exogenous gene in the genome of these each transgenic line.

### Confirmation of Insertion Sites and Flanking Sequences by PCR Amplification and Sequencing

**FIGURE 3:**
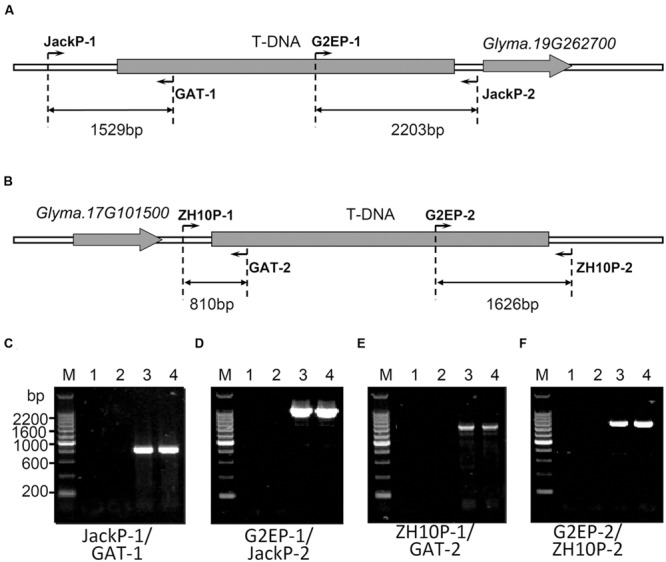
**Locations of primers and PCR validation of transgenic insertion sites.** Schematic diagram of PCR validation primers were designed for GE-J16 **(A)** and ZH10-6 **(B)**. *Glyma.19G262700* and *Glyma.17G101500* were genes located near the insertion sites of two transgenic events. PCR amplifications of junction sequences were carried out in GE-J16 **(C,D)** and ZH10-6 **(E,F)**. M: 200 bp DNA Marker, 1: negative control of water; 2: negative control of non-transgenic soybean Jack **(C,D)** and ZH10 **(E,F)**; 3, 4: transgenic plants of GE-J16 **(C,D)** and ZH10-6 **(E,F)**.

**FIGURE 4:**
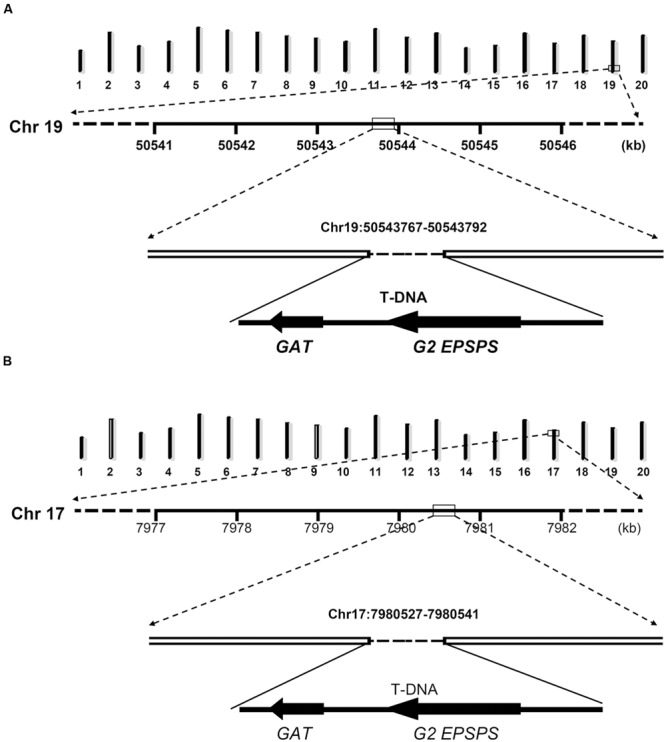
**Schematic diagram of insertion loci and flanking sequences in GE-J16 **(A)** and ZH10-6 (B).** The upper diagram of each chart indicated 20 chromosomes of soybean with chromosome numbers at the bottom. The numbers under the line of Chr 19 **(A)** and Chr 17 **(B)** indicates physical positions of these chromosomes.

In order to characterize exact positions of T-DNA insertions, PCR primers were designed based on speculated upstream and downstream flanking sequences and the T-DNA sequence (**Figure 3**). When using primer pairs with one primer annealing within putative flanking sequences (JackP-1, JackP-2, ZH10P-1, and ZH10P-2) and the other annealing to the exogenous genes (GAT-1, GAT-2, G2EP-1, and G2EP-2), gel electrophoresis revealed that PCR reactions of primer pairs JackP-1/GAT-1, G2EP-1/JackP-2, ZH10P-1/GAT-2, and G2EP-2/ZH10P-2 had generated products with single band in transgenic lines while no product could be detected from the non-transgenic control (**Figure 3**). Sanger sequencing of these junction fragments confirmed the putative insertion sites identified by WGS and exact positions of T-DNA insertions were also identified. The T-DNA of GE-J16 was integrated into physical position 50543767–50543792 on chromosome 19 while that of ZH10-6 was inserted into position 7980527–7980541 on chromosome 17 (**Figure 4**). Both exogenous fragments were all inserted in intergenic regions of the host genome and no functional gene was interrupted by T-DNA insertions. Accordingly, due to the transformation, 24-bp and 13-bp fragments of host genome sequences were replaced by insertions of T-DNA in GE-J16 and ZH10-6, respectively.

### Validation of Insertion Sites in Heterozygous Lines and Segregation Populations

**FIGURE 5:**
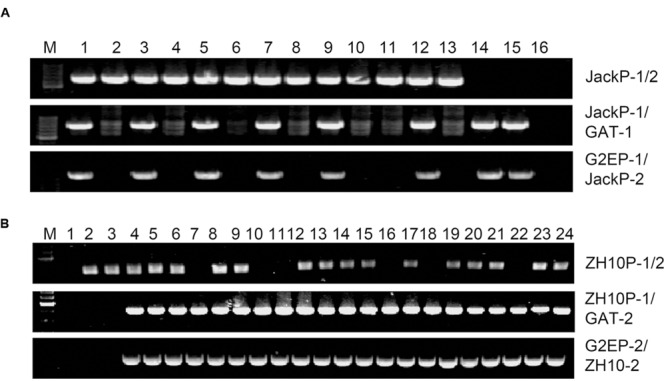
**Validation of the insertion sites in individuals of heterozygosis lines and segregation populations. (A)** Validation of the insertion site in progeny of heterozygosis GE-J16. M: 200-bp DNA marker; 1, 3, 5, 7, 9, 12, 14, 15: glyphosate tolerant individuals; 2, 4, 6, 8, 10, 11, 13: glyphosate sensitive individuals; 16: negative control of water. **(B)** Validation of the insertion site in segregation populations derived from ZH10-6. M: 200-bp DNA marker; 1: negative control; 2,3: glyphosate sensitive individuals; 4–24: glyphosate tolerant individuals.

In order to further validate the insertion sites, specific primer pairs for each event were applied to identify genotypes of individual plants from T_2_ and F_2_ populations. Genomic DNA isolated from random selected glyphosate tolerant and sensitive plants was used as template for PCR amplification. For primer pairs (JackP-1/GAT-1, G2EP-1/JackP-2 for GE-J16 and ZH10P-1/GAT-2, G2EP-2/ZH10P-2 for ZH10-6) amplifying upstream or downstream junction of the plant genome and T-DNA region, expected sizes of PCR products (1529-bp, 2203-bp for GE-J16 and 810-bp, 1626-bp for ZH10-6) were amplified in all glyphosate tolerant plants while no product was detected in all sensitive plants (**Figure 5**), indicating that glyphosate tolerant phenotype co-segregated with T-DNA insertion either in GE-J16 or ZH10-6. For primer pairs JackP-1/2 and ZH10P-1/2 used for amplifying flanking sequences of host genome, expected 1246-bp and 632-bp products were amplified in 13 progeny of heterozygous GE-J16 and 17 F_2_ individuals derived from ZH10-6, respectively. These 30 lines contain heterozygous lines if PCR amplification of upstream or downstream junction sequences could be detected and wild type if junction sequences could not be amplified. In addition, no PCR product of host genome could be detected from two and six glyphosate tolerant plants derived from GE-J16 and ZH10-6, respectively (**Figure 5**). These plants were regarded as homozygous lines since only junctions between T-DNA and host genome could be amplified. Further identification of phenotype in T_3_ generation and F_2:3_ populations also confirmed no segregation of glyphosate tolerant phenotype in these eight lines. This result suggested that the insertion of exogenous genes and glyphosate tolerance phenotype were co-segregated in these segregation populations.

## Discussion

Detailed molecular characterization of inserted DNA and associated flanking sequences is of particular importance in safety assessment of GM crops and in tracing individual transgenic event (Yang et al., 2013). Traditionally, PCR-based methods including TAIL-PCR and genome walking, combined with Southern blot analysis and Sanger sequencing, were applied to determine locations of integration sites and junction sequences between exogenous sequences and host genome (Codex Alimentarius Commission, 2003). However, these methods usually did not work very well in species with relative complex genome. Due to the high level of duplication in soybean, traditional approaches are usually time consuming and their abilities to identify transgenic events are limited by various factors including complex insertion pattern, T-DNA rearrangement, small insertions/deletions and individual nucleotide substitutions. For example, only one copy of *CP4-EPSPS* was initially documented when GM event GTS40-3-2 was approved for commercialization (Padgette et al., 1995). Later, the rearrangement of the 3′-NOS terminator junction and one unintended 70-bp DNA fragment were evidenced (Monsanto Company, 2000; Windels et al., 2001; Wang et al., 2010). Therefore, PCR-based method sometimes may not get complete information of exogenous fragment insertion in transgenic soybeans.

With the emergence and development of high throughput next generation sequencing technology, sequences of whole genome can be obtained rapidly at relatively low cost. NGS has proven to be a powerful tool for discovering genome variation including re-arrangements, gene fusions, DNA structural variations in different species (Campbell et al., 2008; Hormozdiari et al., 2011; DuBose et al., 2013). NGS coupled with bioinformatics platform applied in genomics research are widely used in the agricultural biotechnology field (Kovalic et al., 2012; Lepage et al., 2013; Park et al., 2015). Recently, several researches have focused on new approaches in molecular characterization and safety assessment of transgenic events using NGS technology (Urbanski et al., 2012; Daniela et al., 2013; Pauwels et al., 2015). Here we identified the integrity locations of transgenes and characterized the junction sequences in two newly developed glyphosate-tolerant transgenic soybeans using WGS method. The molecular characterization of these two events at DNA level will serve as risk assessment of them with respect to their possible impact on environment and human/animal health. Even more, this data also provides information for development of detection techniques in tracing these transgenic events.

Compared with traditional PCR-based methods, WGS combined targeted bioinformatics analysis emerge as a sensitive and time- and labor-effective approach in molecular characterization of GM plants. NGS-based molecular characterization can overcome some limitations of PCR-based approaches, including high amount of DNA required, multiple manual work interventions, and the impossibility to identify genetic changes (Pauwels et al., 2015). Particularly, it reduces the cost of experiment and the amount of labor since most of steps can be performed with commercially available kits in high throughput manner (Kovalic et al., 2012). In addition, WGS can further reveal nucleic sequence variations including SNPs and small InDels, which could potentially detect small sequence modifications (Pauwels et al., 2015). Even more, accurate sequence information identified by WGS could be directly used in assessment of the potential toxicity or allergenicity in GM plant by verification of potential similarities in databases of toxins, toxin targets, allergenic proteins and anti-nutritional factors.

Although several NGS based approaches have been developed for molecular characterized of GM plants (Kovalic et al., 2012; Yang et al., 2013; Park et al., 2015), these researches all used paired-end reads with one read of a pair mapped to the transgene and its mate mapped to the plant genome to identify the transgenic insertion. Therefore, the insertion site was just identified as a region as the sequence between read pairs was not sequenced completely. In our analysis, we separated paired-end reads and each one was used for mapping, then one read with a portion derived from the transgene and the other portion derived from host genome was selected for identifying the integration site. In addition, although we achieved lower sequencing coverage (∼20×) compared with previous reports using more than 70× coverage (Kovalic et al., 2012; Park et al., 2015), three out of four junctions could be identified from our single read analysis, indicating the power of WGS method even in species such as soybean with complex genome. Due to the uneven coverage of reads across the whole genome, lower coverage of junction reads was obtained compared to the average sequencing depth. In particular, since the downstream junction of GE-J16 on Chromosome 19 has not been identified, increasing the sequence coverage by deep sequencing is recommended. Nevertheless, the implementation of NGS in GMO routine analysis may be less affordable for some laboratories with modest budgets due to relatively high cost, the requirement of adequate computer infrastructures and qualified analysts in bioinformatics for dealing with enormous amount of sequencing data (Buermans and Dunnen, 2014; Liang et al., 2014; Willems et al., 2016).

## Author Contributions

L-JQ, YG, and BG conceived and designed the experiments. BG, YG, and HH performed the experiments. YG and BG analyzed and interpreted data. BG, YG, and L-JQ wrote the manuscript. All authors read and approved the manuscript.

## Conflict of Interest Statement

The authors declare that the research was conducted in the absence of any commercial or financial relationships that could be construed as a potential conflict of interest.

## Footnotes

1 https://phytozome.jgi.doe.gov/pz/portal.html

## Abbreviations

GMO: genetically modified organism
NGS: next-generation sequencing
PCR: polymerase chain reaction
WGS: whole genome sequencing
